# FROM TALK TO ACTION: POLICY STAKEHOLDERS, APPROPRIATENESS, AND SELECTIVE DISINVESTMENT

**DOI:** 10.1017/S0266462315000392

**Published:** 2015

**Authors:** P. Alison Paprica, Anthony J. Culyer, Adam G. Elshaug, Justin Peffer, Guillermo A. Sandoval

**Affiliations:** alison.paprica@utoronto.ca; Institute of Health Policy, Management and Evaluation, University of Toronto, Health Sciences Building; Menzies Centre for Health Policy, Sydney School of Public Health, The University of Sydney; Ontario Ministry of Health and Long-Term Care

**Keywords:** Health policy, Health economics, Appropriateness, Disinvestment, Evidence-based health care, De-implementation

## Abstract

**Objectives:** There is widespread commitment—at least in principle—to “evidence-informed” clinical practice and policy development in health care. The intention is that only “appropriate” care ought to be delivered at public expense. Although the rationale for an appropriateness agenda is widely endorsed, and methods have been proposed for addressing it, few published studies exist of contemporary policy initiatives which have actually led to successful disinvestment. Our objective was to explore whether the direct involvement of policy stakeholders could advance appropriateness and disinvestment.

**Methods:** Several collaborative engagements with policy stakeholders were undertaken to adapt and combine conceptual and empirical material related to appropriateness and disinvestment from the literature to create tools and processes for use in Canada and the province of Ontario in particular.

**Results:** By combining inputs from the literature with colloquial evidence from policy stakeholders, a definition of appropriateness was developed and, importantly, endorsed by all the provincial and territorial ministers of health in Canada. Second, a reassessment framework was successfully implemented for identifying priorities for selective disinvestment.

**Conclusions:** When scientific evidence was combined with colloquial evidence from policy stakeholders, progress was made on the design and successful implementation of policies for appropriateness and disinvestment.

There is unprecedented interest in decreasing the frequency of unnecessary medical tests and procedures. International examples include the Choosing Wisely campaign (1), the United States Preventative Services Task Force (USPTF) grade C and D recommendations (2), the National Institute for Health and Care Excellence (NICE) “do not do” resources (3), and Australia's federal Quality Management Framework of the Medicare Benefits Schedule (4). In Canada, Health Quality Ontario has an “appropriateness initiative” (5), Choosing Wisely Canada has been launched (6) and appropriateness has been identified as a national priority by Canadian provincial and territorial premiers (7). Notwithstanding the numerous “lists” that identify potential low-value services, prioritizing and implementing disinvestments from within those lists still pose challenges (8–13). Political and social factors can be barriers to evidence-informed disinvestment—once a product or service is funded, withdrawing support for it, wholly or in part, is much harder than deciding not to support it in the first place. Expert and professional opinion, political judgment, the interpretation of local values and traditions, and views from stakeholders are all factors that inform and constrain decision making (13–15). Although the literature generally supports stakeholder “ownership” of policy decisions (14–18), few publications detail *how* policy stakeholders should be involved and *what*, specifically, they can contribute. Our objective was to explore the involvement of policy stakeholders in the design and implementation of tools and processes related to appropriateness and disinvestment in Canada, and the province of Ontario in particular, and to assess their effectiveness.

## METHODS

The authors performed a literature review and engaged with health technology assessment (HTA) experts to identify literature related to appropriateness and disinvestment. The general method was then to engage with Canadian policy stakeholders, primarily government staff but also HTA experts and clinicians with policy (nontechnical/clinical) influence, regarding the best ways of adapting and combining ideas and evidence from the literature to create tools and processes that would be directly useful in defining appropriateness and enabling disinvestment. Government staff (rather than independent researchers) served as knowledge brokers with the role of summarizing and presenting research evidence to groups of policy stakeholders. In engaging with policy stakeholders, the knowledge brokers emphasized an intention to combine technical concepts and empirical findings as reported in the literature with the stakeholders’ understanding of local contexts, their judgments of feasibility of implementation and their policy values.

Regarding “appropriateness,” the process began with a preliminary definition drafted by the authors and based on a World Health Organization report (19) and concepts from Health Quality Ontario (5). The Health Support Committee is a network of Canadian policy staff through which provincial and territorial ministries collaborate and share information. The preliminary definition of appropriateness was sent to fifty-three government staff members of the Health Support Committee and an additional seventeen stakeholders (some individuals and some organizations) with potential contributions to make to policy. These included the Canadian Medical Association's affiliated societies and other medical/professional organizations and associations. The definition was refined based on suggestions provided by twenty-eight respondents representing seventeen professional healthcare provider associations and eleven provinces and territories. It was then brought to the national Health Care Innovation Working Group, co-chaired by two provincial premiers and comprising all provincial and territorial ministers of health, who endorsed it in March 2013.

The Ontario “reassessment framework” was based on 16 criteria proposed by Elshaug et al. (20) The authors worked with an ad hoc internal ministry committee specifically created to address disinvestment, with additional advice from local external clinical and HTA experts, to select a subset of the Elshaug criteria and refine them to serve as “triggers” for identifying priorities for reassessment among existing funded technologies and services in Ontario. The ad hoc committee had responsibility for applying the triggers to identify priority candidates for reassessment, and communicating information about specific reassessment candidates to the policy managers and staff who would have responsibility for acting on them.

## RESULTS

The definition of “appropriateness” was agreed by Canadian provincial and territorial ministers of health in March 2013:

In the context of health care, appropriateness is the proper or correct use of health services, products, and resources. Inappropriate care, in contrast, can involve overuse, underuse, and/or misuse of health services, products, and resources.

Appropriateness is primarily determined by analyses of the evidence of clinical effectiveness, safety, economic implications, and other health system impacts.

The practical application of appropriateness is made when these analyses are qualified by (a) clinician judgment, particularly in atypical circumstances, and (b) societal and ethical principles and values, including patient preferences.

The Ontario reassessment framework was established as a set of seven criteria (triggers) adapted from the sixteen criteria proposed by Elshaug et al. (20) Local policy stakeholders identified triggers that (a) would identify reassessment candidates which would be considered valid from the policy-maker perspective and (b) were implementable with existing data sources in Ontario. Under the Ontario reassessment framework, priority candidates were those that met two or more of the following seven triggers (some of which have the quantitative requirements indicated in brackets): (trigger 1) an evidence-based recommendation against use by an external body; (trigger 2) nominated by a local clinical expert; (trigger 3) safety concerns noted in the literature; (trigger 4) regional and/or temporal variation suggesting inappropriate use; (trigger 5) change likely to provide benefit to a significant number of people in Ontario (criterion: change would have positive impact for least 1,000 individuals per year); (trigger 6) change would be cost saving (criterion: change would result in cost savings of at least $1 million per year); (trigger 7) data suggesting that a significant percentage of patients received inappropriate services or products (criterion: no threshold set, but a working limit of not less than 10% of relevant patient cases may be reasonable, except in cases where clinical experts specify a different limit).

The application of the Ontario reassessment framework did not require information about every trigger for each possible candidate. The emphasis was on gathering sufficient rather than complete information to determine whether further assessment was warranted. The framework documents clearly stated that absence of evidence related to a trigger was not to be interpreted as evidence that the trigger was not met.

The Ontario reassessment framework identified candidates that have since proceeded to selective disinvestment, resulting in an estimated $59 million per year (CDN$) freed up for investment elsewhere. For example, partial disinvestment has been implemented in Ontario for routine vitamin B12 testing, routine ferritin testing, and daily use of diabetes test strips by people with diabetes who do not take insulin. Each of these practices was a priority for reassessment based on triggers 1 and 6 of the reassessment framework and, after further involvement of local stakeholders (without additional external context-free scientific evidence), selective disinvestment was implemented.

The Ontario reassessment framework was also used by the ad hoc ministry committee for disinvestment to identify priority candidates from a list of potential low value practices in Australia (21). Of 156 items on the Australian list, 16 were readily identified as potential Ontario priorities: twelve were flagged by Choosing Wisely, USPSTF and/or NICE recommendations (trigger 1), four others were identified as potential priorities by internal ministry clinical experts (trigger 2). Of these sixteen items, nine had been assessed or addressed in Ontario before the creation of the ad hoc committee and did not require further committee attention. All available trigger information for the remaining seven candidates was brought forward for consideration by the ad hoc ministry committee.

Three of the seven practices related to diagnostic imaging in children. Based on their presence on the Australian list of low value or harmful practices (trigger 1), regional variation with no apparent justification (trigger 4) and possible safety issues related to unnecessary medical radiation (trigger 3), reassessment was recommended for these practices, and is now in progress.

The remaining four practices related to cancer screening or treatment. In each case the literature suggested that a practice was appropriate only for specific risk groups. The internal committee identified unexplained regional variation (trigger 4) but did not have access to risk information for Ontario patients receiving the practice. The committee consequently referred the reassessment candidates to another technical group for additional research.

## DISCUSSION

Significant challenges affect the implementation of disinvestment. These include political and social barriers which require the involvement of policy stakeholders for resolution. Lomas et al. describe three different categories of evidence: medical effectiveness research (context-free scientific evidence); social science-oriented research (context-sensitive scientific evidence); and the expertise, views, values, and realities of stakeholders (colloquial evidence) (15). The three categories are not mutually exclusive as each has a role to play in producing evidence-based guidance for the health system (15). The paucity of examples of successful disinvestment, despite supporting scientific evidence, suggests that colloquial evidence has a major influence, and much literature suggests the importance of colloquial evidence as complementary input if HTAs are to have their intended impact (14;22;23). As described below, the involvement of policy stakeholders, and the combination of their colloquial evidence with scientific evidence, assisted the development of tools and processes related to appropriateness and disinvestment in Canada and Ontario.

The revisions to the appropriateness definition that were made based on policy stakeholders’ input, as shown in capital letters below, clearly reflect colloquial evidence including values, pragmatics, resources, and professional experience and expertise.

IN THE CONTEXT OF HEALTH CARE, appropriateness is the proper or correct use of health services, products AND RESOURCES. Inappropriate care, in contrast, can involve overuse, underuse and/or misuse of health services, products AND RESOURCES.

Appropriateness is primarily determined by analyses of the evidence of CLINICAL effectiveness, SAFETY, economic implications, and other health system impacts.

The PRACTICAL APPLICATION of appropriateness is made when these analyses are qualified by (A) CLINICIAN JUDGMENT, PARTICULARLY IN ATYPICAL CIRCUMSTANCES AND (B) societal and ethical principles and values, INCLUDING PATIENT PREFERENCES.

Notably, the finally agreed definition of appropriateness integrated each of the three categories of evidence: clinical effectiveness evidence was a form of context-free scientific evidence, economic implications represented context-sensitive scientific evidence, and clinician judgement and patient preferences were examples of colloquial evidence.

The triggers of the Ontario reassessment framework, which were selected through deliberation with local policy stakeholders, also included each of the three categories of evidence. Trigger 1, “an evidence-based recommendation by an external body,” will often involve consideration of context-free scientific evidence from other jurisdictions. Trigger 6, “change would be cost saving,” prompts consideration of context-sensitive scientific evidence. Trigger 2, “nominated by a local clinical expert,” may be exclusively evaluated based on colloquial evidence, for example, reflecting clinicians’ professional experience/expertise.

Local stakeholder involvement was essential for successful implementation of selective disinvestment. For example, the Ontario reassessment framework identified vitamin B12 and ferritin laboratory testing as reassessment candidates in November 2011. Implementing changes, however, involved months of work including validation by local HTA experts (24) and government staff working with a table of stakeholders from the Ontario laboratory sector to identify the best implementation option—removal of the check boxes for those tests from the standard laboratory test ordering form.

Policy stakeholder input also contributed to a decision not to disinvest. One priority for reassessment was identified based on published evidence that the practice was of low value; however, given the absence of evidence of harm in the literature, and local policy stakeholder advice that the practice was provided infrequently for a small number of people in Ontario, reassessment and selective disinvestment were not pursued.

The mechanisms and processes for obtaining policy stakeholder contributions were also important. For example, each of the first five priority candidates identified by the reassessment framework were already being considered for reassessment and/or disinvestment at some level within government departments, but had not been acted on. Collectively, the formation of the *ad hoc* committee, the provision of context-free scientific evidence related to it, opportunities for group discussion and a high degree of agreement between the internal ad hoc ministry committee members all strengthened committee members’ resolve and interest in specific disinvestment opportunities.

Although the ad hoc committee on disinvestment was initially formed to respond to a “push” of research evidence from the authors (25), it has been succeeded by a standing ministry committee that exercises a “pull” for research evidence on a regular basis.

## CONCLUSIONS

Scientific evidence combined with and qualified by colloquial evidence from policy stakeholders was a recurring theme: (i) A definition of appropriateness was developed by using policy stakeholder input to modify and adapt text from the literature—the result was a definition which included explicit reference to including colloquial evidence in determining appropriateness. (ii) The Ontario reassessment framework was developed through work with local policy stakeholders to adapt a framework from the literature for Ontario—the result was a framework which included criteria based on colloquial evidence. (iii) Implementation of selective disinvestment integrated scientific evidence with colloquial evidence in the forms of practical advice and values inputs from local policy stakeholders.

By using a method that combined and qualified scientific evidence with colloquial evidence from policy makers, progress was made on appropriateness in Canada and Ontario. Although the definition of appropriateness and the design of the reassessment framework were specifically tailored for the Ontario and Canadian context, the fact that these arrangements succeeded in integrating policy-makers' colloquial evidence and have been successfully implemented, may make them a good starting point for related work in other jurisdictions and settings.

## CONFLICTS OF INTEREST

Dr. Elshaug reports grants from The Commonwealth Fund, during the conduct of the study; personal fees from NPS MedicineWise, grants from HCF Research Foundation, grants from National Health and Medical Research Foundation (NHMRC), other from Cancer Australia, personal fees and other from Capital Markets Cooperative Research Centre, personal fees from Australian Commission on Safety and Quality in Health Care, outside the submitted work.
